# Hereditary renal cancer patient and public involvement group: A collaborative, consensus decision process to develop a communication tool for patient use

**DOI:** 10.1017/cts.2023.39

**Published:** 2023-03-27

**Authors:** Ellen Colvin, Stephanie Ng, John Hepworth, Janice Hepworth, Thomas Hartley, Nicola Godfrey, Karen Tricker, Jeanette Rothwell, Glenda Beaman, Emma R. Woodward

**Affiliations:** 1 Manchester Centre for Genomic Medicine, St. Mary’s Hospital, Manchester University NHS Foundation Trust, Manchester, UK; 2 Faculty of Biology, Medicine and Health, University of Manchester, Manchester, UK; 3 VHL UK/IrelandCharity, Nottinghamshire, UK; 4 Research & Innovation, Manchester University Hospital NHS Foundation Trust, Manchester Academic Health Science Centre, Manchester, UK

**Keywords:** Early cancer detection, patient and public involvement, patient information sheet, hereditary renal cancer, renal cell carcinomas

## Abstract

Patient and public involvement (PPI) must be more frequently embedded within clinical research to ensure translational outcomes are patient-led and meet patient needs. Active partnerships with patients and public groups are an important opportunity to hear patient voices, understand patient needs, and inform future research avenues. A hereditary renal cancer (HRC) PPI group was developed with the efforts of patient participants (*n* = 9), pooled from recruits within the early detection for HRC pilot study, working in collaboration with researchers and healthcare professionals (*n* = 8). Patient participants had HRC conditions including Von Hippel–Lindau (*n* = 3) and Hereditary Leiomyomatosis and Renal Cell Carcinoma (*n* = 5), and public participants included two patient Trustees (*n* = 2) from VHL UK & Ireland Charity. Discussions among the enthusiastic participants guided the development of a novel patient information sheet for HRC patients. This communication tool was designed to aid patients when informing family members about their diagnoses and the wider implications for relatives, a gap identified by participants within group discussions. While this partnership was tailored for a specific HRC patient and public group, the process implemented can be employed for other hereditary cancer groups and could be transferable within other healthcare settings.

## Background and Introduction

Patient and public involvement (PPI) throughout clinical research is vital, especially where these groups become the primary service users of novel translational research outcomes implemented within care pathways. Patient voices are important to guide clinical research strategies using patient’s experiential perspectives. The benefits of public and patient involvement are well-established in the UK and across many countries [1,2]. Patient advocates and PPI groups are useful resources for engaging with patients to understand what they feel is missing in their care, and this co-creation of quality research ensures translational care outcomes are patient-led [1]. In the context of cancer, cancer patients acting as representatives have mirrored these benefits in guiding research agendas and in implementing strategies and outcomes in real-world settings, including within the development of patient education tools specific to cancer [1,3]. Previous studies examining cancer PPI have focused largely on those with a cancer diagnosis at or before the time of their involvement, with issues related to patient cancer progression and dropouts resulting from cancer illness often cited [1,3]. While important, there is missing literature focusing on patient involvement in the context of hereditary cancer where carriers may or may not have a cancer diagnosis and are often subject to intensive early detection surveillance programs. We know hereditary cancer patients have important insights for communicating risk and education tools, and thus [4], PPI groups with these hereditary cancer patients could be a useful tool to better patient outcomes and education.

The International Alliance for Cancer Early Detection (ACED) group is a Cancer Research UK-funded partner initiative involving teams from the Canary Center at Stanford University, the University of Cambridge, the Knight Cancer Institute at Oregon Health, and Science University, University College London, and the University of Manchester [5]. The ACED research initiative is working to improve rates of cancer early detection through advancing technologies to overcome current existing difficulties in this area [5]. Early detection is important for earlier cancer diagnoses which can benefit resultant prognoses.

One pilot study currently underway as a branch of this ACED research initiative is focusing on improving Early Detection of Hereditary Renal Cancer (HRC) patients, also known as the ELECTRIC study [6]. More specifically, the study is trying to address a pertinent issue concerning detection for the most common type of adult renal tumors, renal cell carcinomas (RCCs). Typically, RCCs are detected at an advanced stage which consequently results in worsened patient outcomes, despite recent advances with targeted therapy options for treating RCCs. Around 3–5% of all RCC are caused by a familial germline pathogenic alteration [7]. Various germline alterations associated with a high risk of developing RCC are each also a part of wider conditions including Von Hippel–Lindau disease (VHL), Hereditary Leiomyomatosis and Renal Cell Carcinoma (HLRCC) [7], Birt–Hogg–Dubé syndrome, Hereditary Papillary Renal cancer, and Cowden Syndrome [8]. The ELECTRIC study’s aim is to determine if platelet transcriptomic signatures can be used as an additional surveillance tool in conjunction with standard clinical imaging surveillance to identify early-stage RCC in this cohort of patients with high-risk RCC predisposition to improve patient outcomes [6].

Results from the ELECTRIC pilot study are expected in 2023; however, a HRC PPI group has been created to contribute to the research. PPI group discussions have been drawn on to inform novel avenues for exploration and have guided the development of a novel patient information sheet (PIS) for HRC patients, through the collaborative efforts of both the PPI participants and researchers. Notably, this PIS was designed with the intended purpose to be used by patients as a communication tool to aid in discussions with other family members surrounding their diagnosis and the wider implications of this for relatives. Here, the process of developing the PPI group and creating the PIS resource is discussed further in detail.

### Approach

Developing this HRC PIS for families utilized consensus decision-making in a staged, collaborative process and followed the UK INVOLVE guidance for PPI using consultation, collaboration, and co-production [9]. Initially, the aim for establishing a PPI group forum for these HRC patients was to allow open discussion of their experiences and care to identify any gaps that could be improved upon for future patients and may be useful avenues to explore going forward in research. As a result of initial PPI group discussion, a secondary aim arose to develop a PIS as a communication tool to be used by patients with easily accessible language.

### Development

#### Developing PPI groups

The overwhelming commitment and enthusiasm of the hereditary RCC patients wanting to participate and contribute to the ACED-funded ELECTRIC pilot study led to the formation of the group forum. Patient discussion and feedback, of both clinical stories and experiences being involved in research, was encouraged to allow a safe space to share stories, to gauge motivations for taking part in research, and to inform patient-identified gaps for avenues to improve care and research for future RCC patients. Participants were offered monetary compensation for their time, in line with INVOLVE’s guidelines [10].

The first participant discussion group was invited from the cohort of ELECTRIC study participants, with a range of RCC-associated conditions, to join a virtual participant feedback forum. All ELECTRIC study participants at that time (*n* = 11) were first contacted via phone by the study Research Practitioner to determine initial interest and all were keen to take part. Participants were initially sent a forum-specific invitation letter with a reply slip, an imagery consent form (for using images at conferences and presentations) and an SAE through the post. If these were returned, a follow-up email was sent to those who agreed to take part for session details with a further follow-up phone call to ensure patient attendance and to build rapport. The first patient group consisted of four female participants from England, all with HLRCC. To structure the session, answer patient questions, ask follow-up questions, record discussions, and ensure patient safety, a small team of healthcare professionals (HCPs) and researchers also attended the session. The total workgroup for the first session thus consisted of RCC patients (*n* = 4), research practitioner (*n* = 1), consultant clinical geneticist (*n* = 1), research assistant (*n* = 1), clinical trials manager (*n* = 1), and the project coordinator for the ACED ELECTRIC study (*n* = 1).

All ELECTRIC study participants at that time (*n* = 14) were invited to participate in the second PPI group session, including those who attended the first session but excluding those who rejected the first PPI group invitation (*n* = 2). Furthermore, invites to take part in the second PPI group session were sent to patient Trustees (*n* = 2) from VHL UK & Ireland Charity (one of whom was already as participant within the ELECTRIC study) to widen the representation of the PPI group. All participants invited to be a part of the second PPI group to review the PIS and for further group discussion were sent the completed PIS draft to give time to review this prior to the second group meeting for verbal review and discussion. This was also carried out to allow those who wanted to provide feedback and contribute to the PIS, the opportunity to do so in the form of written feedback, if, for any reason, they could not or did not want to attend the PPI second virtual session. The second session consisted of a total of 5 participants and patient advocate members, including participants with VHL (*n* = 3) and HLRCC (*n* = 1), plus one participant’s partner both of whom were advocate Trustees from the VHL UK/Ireland Charity. Additionally, the second group included a consultant clinical geneticist (*n* = 1), clinical trials manager (*n* = 1), research genetic counselor (*n* = 1), and the project coordinator for the ACED ELECTRIC study (*n* = 1). This mixed group approach for both sessions facilitated patient-led discussion and feedback in a semi-structured manner, using prompting open-ended questions (Appendix 1), and allowed a mix of academic, clinical, and participant perspectives to contribute to each session.

#### Patient information sheet initial development

Discussion among participants within the first PPI group for the ELECTRIC study emphasized common shared experiences, which led to emerging themes. One dominant theme that emerged was related to participants’ experience of struggling to relay important disease-related information to their relatives. This difficulty manifested itself through issues in relaying complex genetic information and in conveying the significance of diagnoses and related implications for other at-risk family members. Additionally, it was felt there was a need for general practitioners (GPs) to be more informed about the HRC conditions as they are first point of contact for patients and the first to give disease-relevant information. On the back of this theme, patients suggested a PIS would be useful as a communication tool to pass on to family members to overcome difficulties in relaying disease-related significance and information. This was a welcomed unexpected outcome from the session that could be materialized quickly to address the need.

The first draft of the PIS was drawn up using plain English based on a qualitative review of a variety of patient-facing materials deployed in the Manchester Centre for Genomic Medicine clinic including approved plain language genetic information sheets for other hereditary conditions, such as Lynch syndrome. The PIS was further cross-referenced against other patient information websites, charity information pages, and resources to ensure accurate information with easy readability was achieved for the first draft [11–13].

#### Review and consensus discussion

The PIS draft was first reviewed in a virtual meeting with only clinical team members and researchers who attended the PPI group session to ensure a secondary check for accuracy of clinically relevant information, with edits made throughout the draft after coming to an HCP group consensus decision. Further to ensuring accuracy in the clinical information, this initial HCP-only meeting was important to ensure the PPI review in the second group session could be focused on participant perspective within a reasonable session length to allow patient voices and feedback to be heard but avoid taking too much participant time.

The second PPI group meeting utilized participant consensus-driven decisions to drive the editing process and finalizing of the PIS. Each section of the PIS was read in turn to the PPI group by a researcher with time to reflect and give feedback after. Consensus was achieved when all participants and researchers within the PPI group agreed in a section-by-section manner upon the content readability, accessibility, and how well the information addressed the missing need initially highlighted from the initial PPI group. Participant discussions gave both positive and negative feedback and finalizing sessions and reaching consensus involved discussion of raised issues, adding, or editing consequential changes, and all participants and researchers unanimously agreed upon the section as accepted (Table 1). This process was easily generated and would be easily reproducible for other patient groups.

**Table 1.** Themes identified from second patient and public involvement (PPI) group review of the patient information sheet (PIS) and the associated amendments derived from PPI participants to improve the communication tool for hereditary renal cancer (HRC) patient relatives

Themes of second PPI group review and feedback of PIS	PIS amendment
Lacking recognition of the complexity of the journey to diagnosis for patients in the PIS	Added sentence to PIS: “Often for many individuals and families, it can take some time for health professionals to recognize that the collection of features and symptoms are due to an underlying hereditary diagnosis because these syndromes are rare in the population”
Use of inaccurate, misleading language about hereditary renal cancer surveillance strategies	Changed the phrase “preventing kidney cancer” in the PIS, used to describe the importance of kidney cancer surveillance substituted for “potentially preventing advanced kidney cancer,” based off a participant’s suggestion.
Defining “surveillance”	Glossary added to the PIS to better define the term “surveillance” after group suggestions to further clarify the meaning for people who may have less familiarity with this term in this context, as likely recipients of this PIS.
Missing resources	Addition of two useful resources to the useful links section in the PIS, after suggestions from two participants to increase the resources available to family members.

One common theme highlighted among several participants was the lacking information in the PIS that gave recognition to the complex journey of getting to a final diagnosis, often called the diagnostic odyssey [14; Table 1], which frequently occurs for HRC conditions and other rare diseases. This experience sparked further discussion, and similarities were shared by others in the group. Based off this participant’s suggestion, a useful sentence was added to address this missing recognition and to acknowledge this common difficulty felt among these patients (see Table 1).

Consideration was additionally given to the use of the phrase “preventing kidney cancer” in the PIS, used to describe the importance of kidney cancer surveillance (see Table 1). This was felt to be misleading among multiple participants because there remains a chance that renal cancer could develop. The point was raised that added surveillance was to detect the cancer earlier, a stark difference from preventing renal cancer development altogether. This sentence was changed based off a participant’s suggestion (see Table 1). The change was strongly agreed upon from all participants and researchers within the workgroup.

A further phrase discussed after prompts from researchers was the term “surveillance” to describe the monitoring of patients to detect any abnormalities. This phrase is used by cancer medical HCPs in academic writing but may not have the same interpretation or meaning for patients. While participants were happy with the understanding of the term, a glossary was added to the PIS after group suggestions to further clarify the meaning for people who may have less familiarity with this term in this context (see Table 1). Within both PPI group meetings, experiences, perspectives, and feedback was recorded by two research team members summarizing keynotes for these sessions.

Furthermore, two additional useful links were added to the useful links section after suggestions from two participants increasing the resources available to family members (see Table 1). More specifically, this included adding a useful free download link to a VHL information handbook resource created by VHL Alliance [15].

#### Additional feedback and discussion

Written feedback was largely positive from participants who reviewed the PIS and gave their responses via email as opposed to attending the virtual PPI group review session. Comments focused on its readability with the PIS being reviewed as clear, informative, well-written, and understandable. One comment criticized its repetitiveness in some sections, and this was further reviewed by the research team when looking at the final draft.

Interestingly, after prompting questions from researchers asking how participants felt about current early detection surveillance imaging and other early detection mechanisms, participants' discussion illustrated the mixed emotions and experiences among the group toward ED. One participant outlined he was happy attending current standard surveillance imaging, while another described the diagnosis of his HRC condition was later in his life and he would not have wanted to have annual screening any earlier despite his right kidney cancer being detected at a later stage. This point emphasizes the importance of not overdoing the frequency of ED surveillance and ensuring patients still feel they can lead a normal life without being subject to unnecessarily large amounts of time under surveillance. Another participant highlighted they would want ED surveillance and testing for a condition from a younger age than is currently offered and her struggle with getting genetic testing and imaging surveillance for her children. Concluding on that discussion, one participant highlighted that while ED imaging is good once you have a diagnosis, issues of the diagnostic odyssey and getting to that stage need more focus.

## Discussion

This PPI group represents a shared interest and agenda from both HCPs and patients. This mutually beneficial group enables participants to have a safe space to talk, to share their stories and their needs for future care and research, allowing HCPs and researchers to utilize this information to ensure research is patient-led and fulfills the needs of those primary patient group service users. From this PPI group, the resulting unexpected but vital material outcome was a communication tool in the form of a PIS for patient use.

### Lessons from the PPI group sessions

The infrastructure of the PPI group having both researchers and patients was instrumental to the working of the PPI and to the PIS material outcome. Both working PPI group sessions had at least 40% patient representation ensuring the basis of all discussions was underpinned with a patient-first, patient-led agenda. The HCP involvement helped to facilitate the session, to ensure patient safety, and to take notes for directing for future research to improve patient care. Involvement of the researchers and HCPs within this PPI group further was essential to drafting up the PIS and organizing PPI group feedback and involvement. Sending this PIS out prior to the second PPI group session worked well allowing participants to properly assimilate the information in their own time, before giving their feedback.

HCPs provided open-ended questions helping to create the semi-structured approach and encouraging active interactions for participants’ PPI group discussion, within- and between-HCP and patient groups. Considering this further, it was easy for discussion to deviate from the initial focal topic of these questions. Participants were able to positively ascertain and consolidate their agenda and needs, and openly communicate this back to researchers and HCPs ensuring a participant-led format. This was exemplified here with the unexpected need for the communicative PIS tool that was established in PPI group discussion and later materialized. With this said, if researchers are wanting to gain a more direct insight into patient responses with more focused, specific answers, this may not be the preferred approach. Utilizing a mixture of closed- and open-ended questions could be optimal to facilitate both more focused answers and more open discussions in future PPI group sessions. Additionally, ascertaining patient understanding of questions may have helped to focus discussion.

A major theme of both PPI group sessions was the sharing of experiences and complexities of personal care pathway stories among participants. PPI group sessions created a safe space for these participants to be vulnerable and are to continue as a 6-monthly recurring PPI group meeting. This may highlight the need for either additional support groups or easier access and better signposting to those already available for these HRC patient groups. Furthermore, more HCP involvement within these support groups or more opportunity for HCP-patient contact time may be an important takeaway where participants were able to get answers to many of their own questions. PPI sessions empowered participants through increased disease awareness and understanding from both HCP involvement and participant-shared experiences, which may have been missed without these sessions.

The inconsistency in participants who attended both the first and the second session may have slowed the process of the second session where explanations were required to inform patients of the process so far and for repeated introductions for all patients. With this said, the variety of participants attending both sessions helped to reaffirm there was a need for this missing PIS tool for this patient group, where all participants from both groups agreed upon its importance with those second PPI group attendees demonstrating their appreciation for its development. A larger, more diverse expansion of participants in the future would help to increase PPI group representativity of the HRC patient population.

It is important to note that this HRC PPI group may have been more significant within this family cancer setting because of the emotional burden that can accompany HRC, such as with other hereditary conditions. Concerns for relatives and children can bring along more challenges in addition to the rarity of the disease adding to complexity where HCPs are less likely to encounter more rare diseases. This may have allowed this safe space created for these participants to be more meaningful than in other group settings.

### Limitations

As the initial patient group consisted of only white English national females and there was a limited number of patient participants within this group (*n* = 4), this may limit how representative this first PPI group session was of the patient population. This may be offset by the second reviewal PPI group which included a wider range of ethnicities, sexes, RCC-related conditions, and age groups in its makeup, and patients praised the created PIS and shared this felt need with those from the first PPI group. Furthermore, the small number of total PPI group participants from both sessions (*n* = 9) and the low variety of RCC-related conditions involved (mainly, VHL and HLRCC patients participated) may be reflective of the characteristically small numbers of the HRC population as a group of rare genetic disorders.

One significant issue that occurred was with technical difficulties that prevented one participant from accessing the PPI group. Although this issue is one that is inherent when holding virtual sessions, more preparation for this circumstance would have helped to come up with a fast solution and to allow this participant to participate. Future solutions could include utilizing a face-to-face group to ensure technological difficulties do not hinder participation, enabling a call-in option, if there are issues with accessing the virtual platform or having someone on hand to resolve technical issues. To offset this issue, the option of providing either written or verbal feedback within the PPI session was initially given, and thus, while access to the virtual session was not possible, written participant feedback was still utilized.

More specific to the PIS itself, at present, it is only written in British English language and as such may be less useful to other nationalities where different cultures may have different interpretation of words. Additionally, it will likely require further validation if translated directly to different languages where some genetic terms and jargon have different words/phrases or may not exist cross-culturally. This risk was minimized again by using plain English and including a glossary to explain complex terms but further inclusion of wider communities and countries within the development of the PIS may increase its reach for HRCC patient populations.

## Conclusion

While there are advances within early detection research for hereditary cancer and patient voices are being increasingly heard, there is still further need for more deeply embedding patient input into every stage of research to ensure they, as the primary stakeholders of any care quality improvements or translational research outcomes, have their agenda at the forefront and needs being met. PPI groups are a great tool for embedding patients more firmly within research. This HRC PPI group with participants recruited from the ELECTRIC study demonstrates how listening to patient voices and working together with HCPs and researchers in a healthcare setting can have positive beneficial outcomes. The PIS will now be available for genetics HCPs at Manchester Centre for Genomic Medicine to pass on to HRC patients to aid their communication with their relatives. This both benefits public and patient groups, but also HCPs themselves where sharing this communication tool created from these PPI sessions increases awareness of important ED surveillance that is available and more generally, increases understanding of complex genetic information. This is especially crucial for rare disease groups such as HRC-associated conditions, where smaller disease population numbers may mean less research focus is on these rarer patient groups.

## Supplementary material

For supplementary material accompanying this paper visit https://doi.org/10.1017/cts.2023.39. click here to view supplementary material

## Appendix A

Open-ended questions from PPI group session 1

- How did you feel when you were invited to take part in the ELECTRIC study?
- Why did you agree to take part in the ELECTRIC study?
- What do you foresee the future to bring for people diagnosed with RCC in future generations?

Open-ended questions from PPI group session 2 (after reading through the PIS)

- What your experiences are with GPs?
- Imaging currently is the way we do ED and surveillance, would there be any different means that you would want to have ED surveillance? What does ED mean to you? What ideas for testing would you like for ED?
- What do you think are barriers to participating in research?

*A copy of the patient information sheet is available on request*.
