# Supplementary material for: Hereditary renal cancer patient and public involvement group: A collaborative, consensus decision process to develop a communication tool for patient use
Source: J Clin Transl Sci. 2023 Mar 27;7(1):e115. doi: 10.1017/cts.2023.39 (PMC10225253; doi:10.1017/cts.2023.39)
Supplement: Supplementary file 1 [file S2059866123000390sup001.docx]

**Hereditary kidney cancer: information sheet for families**

**Glossary**

| Surveillance: Monitoring scans and investigations to detect abnormalities at an early stage |
| --- |

You have been given this information sheet because an inherited condition has been identified in your family that could potentially mean you are at increased risk of developing kidney cancer. Early detection strategies are offered to individuals known to be at increased risk.

***What is hereditary kidney cancer?***

Certain hereditary syndromes can increase the risk of developing kidney cancer. In most cases, kidney cancer is not passed down from parent to child. When kidney cancer is seen across multiple generations in multiple different family members, this can indicate there may be an underlying hereditary kidney cancer. Hereditary kidney cancer is rare and accounts for only 5% to 8% of all kidney cancers, and it is usually linked to a hereditary syndrome.

A hereditary syndrome is a set of signs and symptoms or conditions that occur together and is caused by certain genetic alterations that can be passed down from a parent to a child. Hereditary syndromes are sometimes called genetic syndromes or family cancer syndromes. In this information sheet, the hereditary syndromes described all have an associated increased risk of developing kidney cancer. There are several hereditary syndromes which have an associated hereditary kidney cancer risk (see useful links). Some examples of these include:

- von Hippel-Lindau disease (VHL)
- Hereditary leiomyomatosis and renal cell cancer (HLRCC)
- Birt-Hogg-Dubé syndrome (BHD)
- Hereditary papillary renal cancer (HPRC)
- Cowden Syndrome

Each of these syndromes, as well as other hereditary kidney cancer syndromes, will have a different gene that is altered causing the symptoms of the syndrome and an increased risk of developing kidney cancer. Often for many individuals and families, it can take some time for health professionals to recognise that the collection of features and symptoms are due to an underlying hereditary diagnosis because these syndromes are rare in the population.

***How is the gene inherited?***

We all have two copies of each of our genes. An individual who carries a gene change in a gene associated with hereditary kidney cancer also has a normal copy of this gene. When they come to have children, each child will inherit either the normal or altered copy. Each child independently (whether male or female) therefore has a 50% chance of inheriting the altered copy. Now that the specific gene change has been identified in your family, it is possible to find out whether you carry an altered gene for the same familial syndrome.

It is important to remember that not everyone with a gene change for hereditary kidney cancer will develop cancer. Just knowing about the gene change in the family now means we can advise you about surveillance to detect abnormalities, and possibly prevent advanced kidney cancer.

***What surveillance may be available?***

If a genetic alteration is identified in an individual after genetic testing, additional surveillance is offered to help to detect any abnormalities that may develop, early on. Surveillance, which may include annual imaging, is offered to identify changes before any symptoms arise. If any abnormalities are detected early on, we can ensure the best and most appropriate follow-up is carried out. Follow-up can include treatment, removal, further monitoring, or the decision that no further action is required at that time.​

***What to expect from genetics?​***

In clinical genetics, specialist healthcare professionals are trained in rare genetic diseases and conditions and can provide genetic counselling. Genetic counselling can aid the process of understanding of a genetic disease or condition, such as syndromes associated with hereditary kidney cancer, and provide information on all the available options to you to help you to decide which options are the next best steps for you.​

During a genetic counselling session, a person's family and personal medical history may be discussed and if appropriate, may lead to genetic testing.

***What can I do?***

If you would like to find out more about hereditary kidney cancer and what it means for you and your family, please ask your GP to refer you to your local genetics department quoting the reference number at the top of this page. An appointment in genetics will not put you under any obligation to have a genetic test. Just knowing about the gene change in the family means we can still advise you about surveillance and other measures that may be relevant for you.

If you have any concerning symptoms at the current time, please seek advice from your GP in the usual manner.

***Useful links***

VHL alliance: <https://vhl-uk-ireland.org/> [www.vhl.org](http://www.vhl.org)

HLRCC alliance: <https://hlrccinfo.org/>

BHD foundation: <https://bhdsyndrome.org/>

PTEN UK & Ireland Patient Group: <https://ptenuki.org/>
